# Percutaneous Closure Based on Physiological Assessment of an Arteriovenous Fistula in a Patient With Chronic Limb Threatening Ischaemia

**DOI:** 10.1016/j.ejvsvf.2023.11.001

**Published:** 2023-11-10

**Authors:** Keisuke Shoji, Michitaka Kitamura, Shiori Yoshida, Kenshi Ono, Naotoshi Wada, Tetsuya Nomura, Natsuya Keira, Tetsuya Tatsumi

**Affiliations:** Department of Cardiovascular Medicine, Kyoto Chubu Medical Center, Kyoto, Japan

**Keywords:** Arteriovenous fistula, Laser Doppler flowmetry, Chronic limb threatening ischaemia

## Abstract

**Introduction:**

An arteriovenous fistula (AVF) is a potential complication of endovascular therapy (EVT). Arteriovenous fistula steal syndrome sometimes leads to severe limb ischaemia; however, assessment of peripheral perfusion in AVF has not yet been established.

**Report:**

A 90 year old woman diagnosed with chronic limb threatening ischaemia underwent EVT. However, subintimal angioplasty of infrapopliteal lesions resulted in AVF formation in the posterior tibial artery (PTA). Revascularisation of the anterior tibial artery and PTA was performed, but severe AVF steal syndrome persisted and wound healing was delayed. An attempt to physiologically assess the effects of AVF closure and perform an AVF closing manoeuvre, if necessary, was performed. The physiological assessment was performed by laser Doppler flowmetry (LDF) and blood flow was temporarily blocked via the AVF at the distal PTA using a 6 F guiding extension catheter. A significant increase in blood flow was observed in the perfused area of the plantar artery. Coil embolisation and covered stent implantation in the PTA completely closed the AVF. During the procedure, peripheral perfusion with LDF gradually increased in the heel and fifth toe. After AVF closure, the skin perfusion pressure values increased significantly, wound healing was accelerated, and complete healing was achieved.

**Discussion:**

Laser Doppler flowmetry measurements under simulated AVF closure using a guiding extension catheter may be useful for the physiological assessment of peripheral perfusion before percutaneous AVF closure.

## Highlights

• Arteriovenous fistula is a potential endovascular treatment complication that can lead to limb ischaemia.
• Most intra-operative arteriovenous fistula cases can be managed using an endovascular technique.
• The assessment of limb ischaemia caused by arteriovenous fistula steal has not been established.
• Laser Doppler flowmetry may be useful to physiologically assess ischaemia before arteriovenous fistula steal closure.

## Introduction

Endovascular therapy (EVT) is widely performed for limb salvage in patients with chronic limb threatening ischaemia (CLTI). Although EVT is considered safe and less invasive, the development of an arteriovenous fistula (AVF) is a rare complication.^1^

While upper extremity ischaemia due to an AVF resulting from dialysis vascular access is a common occurrence, lower extremity ischaemia due to an iatrogenic AVF is much rarer.^2^ A steal syndrome associated with dialysis occurs when there is excessive blood flow to a low resistance vascular bed (arteriovenous shunt) and insufficient blood flow to a high resistance vascular bed (distal extremity).^3^ This phenomenon can also manifest in AVFs of the lower extremity because many patients with CLTI have poor distal runoff vessels. However, the assessment of peripheral perfusion associated with steal ischaemia has not been established. Therefore, it is often difficult to predict the effectiveness of AVF closure a priori.

This study describes a case of percutaneous closure based on a physiological assessment of steal ischaemia induced by an iatrogenic AVF in CLTI.

## Case report

**Figure 1:**
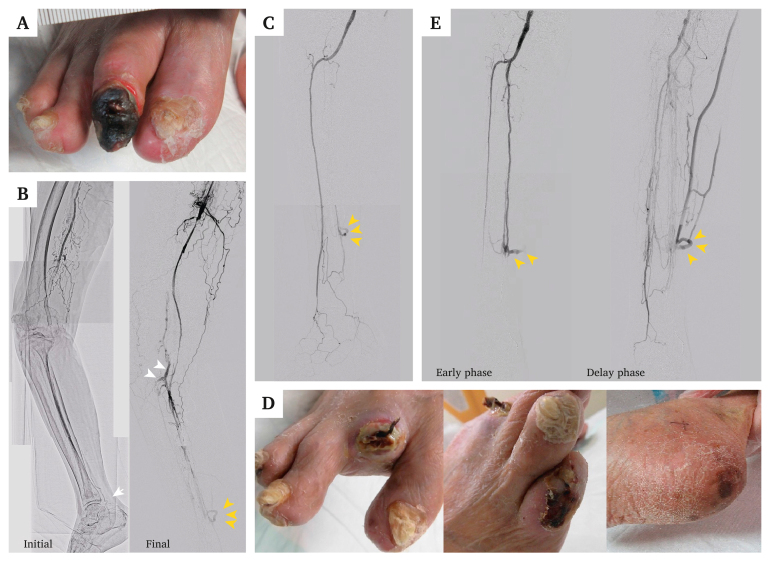
Angiography of an iatrogenic arteriovenous fistula in first to third sessions of endovascular therapy, and delayed wound healing in the right foot. **(A)** Gangrene in the second toe. **(B)** Initial and final angiography in the first session procedure (white arrow = distal puncture point of the distal posterior tibial artery; white and yellow arrowheads = iatrogenic arteriovenous fistula). **(C)** Final angiographic image after revascularisation of the anterior tibial artery in the second session procedure (yellow arrowhead = arteriovenous fistula). **(D)** Delayed wound healing in the second toe and a new wound in the fifth toe and heel. **(E)** Final angiogram after revascularisation of the posterior tibial artery in the third session procedure (yellow arrowhead = severe steal phenomenon through the arteriovenous fistula).

A 90 year old woman with gangrene of the fourth toe in her right foot had been diagnosed with CLTI one year previously. After implantation of two drug eluting stents in the right superficial femoral artery (SFA) and balloon angioplasty in the anterior tibial artery (ATA), the wound healed completely. However, she developed gangrene of the second toe of her right foot one month earlier and was diagnosed with recurrent CLTI (Fig. 1A). The ankle brachial index (ABI) was immeasurable, and the skin perfusion pressure (SPP) was low (21 mmHg at the dorsal and 6 mmHg at the plantar). The patient's foot condition was identified as WIfI stage 4 (wound = 2, ischaemia = 3, foot infection = 1). An initial angiogram demonstrated in stent occlusion in the SFA, and the distal posterior tibial artery (PTA) to the plantar artery appeared as an infra-malleolar vessel runoff (Fig. 1B). Although a 0.014 inch guidewire was advanced into the distal popliteal artery, it entered the subintimal space. Therefore, a microcatheter was retrogradely inserted through the distal PTA and a guidewire was advanced using the looped wire technique, resulting in guidewire externalisation. A 2 mm balloon was dilated in the popliteal artery and PTA; however, angiography showed an AVF in the proximal and distal PTA (Fig. 1B). The first EVT was terminated because improvement in antegrade flow could lead to AVF exacerbation.

Two weeks later, the second EVT achieved successful revascularisation from the SFA to the ATA. However, the dorsalis pedis artery was absent, and the antegrade blood flow was stolen via the AVF at the distal PTA (Fig. 1C). The right ABI rose to 0.5 and only the dorsal SPP value improved (48/9 mmHg); thus, local wound care was continued. However, new wounds appeared on the heel and fifth toe (Fig. 1D).

**Figure 2:**
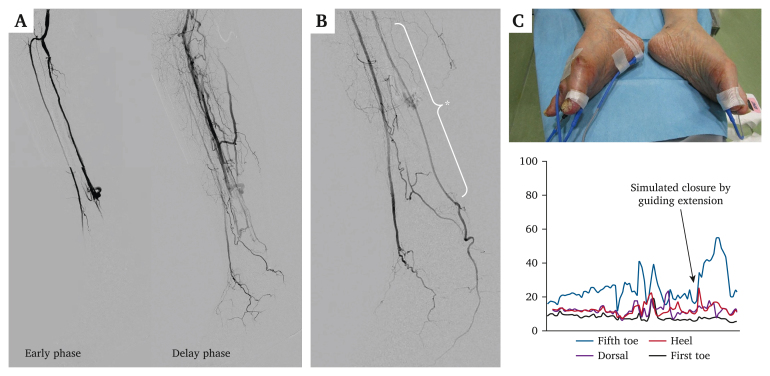
Preliminary physiological assessment of peripheral perfusion via laser Doppler flowmetry. **(A)** Severe steal phenomenon through the arteriovenous fistula on the initial angiogram. **(B)** Simulated closure of the arteriovenous fistula using a guiding extension catheter (asterisk indicates the guiding extension catheter). **(C)** Laser Doppler flowmetry probes attached to the skin on the dorsal, heel, first toe, and fifth toe areas of the foot. Measurements of LDF in the fifth toe increased remarkably after simulated closure of the arteriovenous fistula.

**Figure 3:**
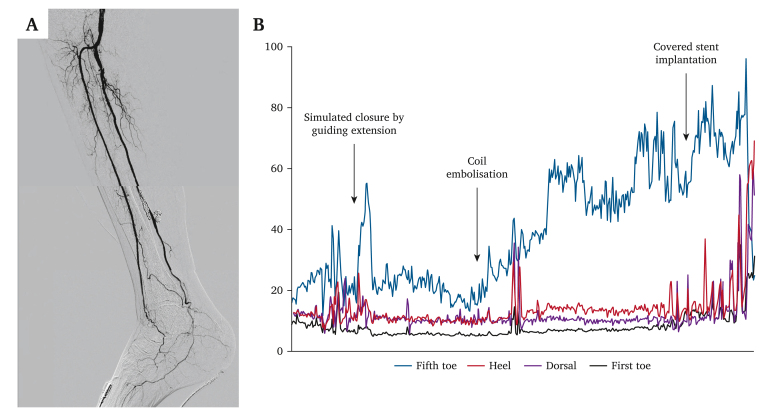
Final angiogram after implanting a covered stent and changes in peripheral perfusion according to laser Doppler flowmetry during the procedure. **(A)** Final angiogram showing complete closure of the arteriovenous fistula and direct flow to the forefoot. **(B)** Changes in laser Doppler flowmetry measurements during the procedure. Peripheral perfusion using laser Doppler flowmetry gradually increased in the heel and fifth toes during coiling and covered stent implantation.

The PTA was successfully re-vascularised in the third EVT session. Nevertheless, severe steal syndrome due to the AVF at the distal PTA persisted despite prolonged balloon inflation (Fig. 1E). Skin perfusion pressure improvement after revascularisation was insufficient (44/13 mmHg) and external AVF compression failed to improve the value. Therefore, it was attempted to physiologically assess the effect of AVF closure and, if necessary, perform an AVF closure with coil embolisation. A physiological assessment of the microcirculation was performed using laser Doppler flowmetry (LDF; JMS Co., Ltd., Tokyo, Japan). The measurements were obtained using LDF probes attached to the skin in the dorsal, heel, first toe, and fifth toe areas. A 4.5 F guiding sheath was inserted through the right common femoral artery and a guidewire was advanced to the plantar artery. The shunt flow was temporarily blocked by placing a 6 F Guidezilla extension catheter (Boston Scientific, Marlborough, MA, USA) across the AVF inlet. The extension catheter is typically employed to provide additional support or facilitate the smooth delivery of devices to the target lesion. Since the lumen diameter of the vessel and the outer diameter of the extension catheter were comparable, the extension catheter was used to reduce the shunt volume of the AVF in this case. A significant increase in blood flow was observed in the heel, especially the fifth toe, which is the plantar artery perfusion area (Fig. 2A–C). Therefore, it was attempted to close the AVF using coiling embolisation. A 3 F guiding catheter was inserted retrogradely via the distal PTA, and two major vein branches were embolised using coils; however, the veins were so markedly branched that it was difficult to achieve complete closure. Finally, a covered stent was implanted in the PTA, achieving complete AVF closure (Fig. 3A). Laser Doppler flowmetry measurements gradually increased in the heel and fifth toe during coiling and covered stent implantation (Fig. 3B). After the procedure, the SPP significantly increased (48/40 mmHg) and complete would healing was observed. No clinical recurrence was observed for 10 months after wound healing.

## Discussion

The main AVF aetiology in lower extremity arteries is traumatic or iatrogenic. The incidence of AVF related to subintimal angioplasty is reportedly 0.8%, suggesting that an AVF is a potential complication of EVT.^1^ A previous study showed that one third of all iatrogenic femoral AVFs related to catheterisation spontaneously closed within 12 months and no signs of leg damage were observed despite the persistent AVF.^4^ Therefore, conservative management is often considered for asymptomatic iatrogenic AVFs. In contrast, invasive management strategies may be necessary for symptomatic AVF such as limb ischaemia. In this case, delayed wound healing and new wounds were observed despite successful revascularisation by a direct straight line from the SFA to ATA. Therefore, the impact of steal syndrome on limb ischaemia related to AVF should have been accurately assessed.

The SPP is useful in assessing peripheral perfusion in CLTI patients. Moreover, SPP was considered useful in assessing ischaemia in the upper extremities caused by arteriovenous access steal in haemodialysis patients.^5^ However, there is no established method for evaluating steal ischaemia. In the present case, an increase in SPP value during manual compressing of the AVF was not observed, probably because of the inability to fully suppress the shunt caused by several branches on the venous side. Therefore, the effect of AVF closure was physiologically evaluated using a simulated shunt closing manoeuvre with the extension catheter and the microcirculation was simultaneously measured with LDF. The PTA lumen diameter was approximately 2 mm, whereas the outer diameter of the 6 F Guidezilla was 1.7 mm; thus, selective insertion into the PTA can reduce shunt flow, leading to simulated AVF closure. In a previous report, AVF by subintimal angioplasty almost always occurred at the infrapopliteal arteries;^1^ therefore, this method could be applicable to evaluating ischaemia caused by AVF following subintimal angioplasty. In this case, it was assumed that the AVF closure promoted wound healing because the LDF measurements significantly increased after the simulated shunt closure. The LDF measurements in the plantar area gradually increased during the closure procedure, the SPP value of the plantar area markedly increased after the procedure, and the wound healed completely.

It is believed that this is the first report to preliminarily evaluate the physiological microcirculation using LDF before AVF closure in the lower extremities. Laser Doppler flowmetry has been used for the early diagnosis of lower extremity artery disease, management of vascular access in dialysis, and detection of distal emboli during EVT.^6^, ^7^, ^8^ Measurements are obtained by attaching the probe to the area to be measured and laser beams are produced by a semiconductor laser diode that is installed in the LDF probes. These beams penetrate the skin, hit red blood cells in the vasculature, and are dispersed. The laser beams are then converted to scattered light by frequency variation, which are recognised as electrical signals by a photodetector. This technique is superior to SPP as it is less invasive without the need for compression with a cuff and can be measured continuously; subsequently, the effect of improved microcirculation during EVT can be seen immediately. Thus, although LDF has high sensitivity in microcirculation assessment, LDF measurements are affected by motion artifacts, ambient temperature variations, and medications caused by the high sensitivity.^9^ To solve this problem, another LDF probe is attached to the contralateral leg, as a control, to determine whether the change in the measurements on the affected side is reliable. In this case, the changes in the measurements at the affected site were deemed reliable because the control LDF measurements were consistently stable during the procedure.

Most infrapopliteal AVFs during EVT can be managed using endovascular techniques (coil embolisation, balloon tamponade, alternative dissection, covered stent placement, and vascular plugs).^1^^,^^2^^,^^10^ Prolonged balloon inflation should first be attempted for AVF closure, but other closure techniques should be attempted if that does not work. In the present case, coil embolisation was selected as an alternative because of its minimal effect on the artery. However, coil embolisation was stopped because of the risk of coil displacement to the arterial side and a covered stent was implanted. Although covered stent implantation is a simple and easy method for AVF closure compared with coil embolisation, the patency and adequate duration of dual antiplatelet therapy should be further investigated.

### Ethics

All procedures performed in this study were in accordance with the ethical standards of the institutional and or national research committee and the 1964 Helsinki Declaration and its later amendments, or with comparable ethical standards. Written informed consent was obtained from the patient for the publication of this case report and any accompanying images and videos. A copy of the written consent form is available for review by the journal editor.

## Funding

Not applicable.

## Authors' contributions

KS managed the patient and wrote the manuscript. All authors read and approved the final manuscript.

## Conflict of interest

The authors declare that they have no competing interests.
